# Lentivirus-mediated PLCγ1 gene short-hairpin RNA suppresses tumor growth and metastasis of human gastric adenocarcinoma

**DOI:** 10.18632/oncotarget.6976

**Published:** 2016-01-22

**Authors:** Bingchang Zhang, Fen Wang, Lianzhi Dai, Heguo Cai, Yanyan Zhan, Song Gang, Tianhui Hu, Chun Xia, Bing Zhang

**Affiliations:** ^1^ School of Medicine, Xiamen University, Fujian, 361102, China; ^2^ Zhongshan Hospital, Xiamen University, Fujian, 361004, China

**Keywords:** PLCγ1, ShRNA, tumor growth, tumor metastasis, human gastric adenocarcinoma cells

## Abstract

Targeted molecular therapy has gradually been a potential solution in cancer therapy. Other authors' and our previous studies have demonstrated that phosphoinositide-specific phospholipase γ (PLCγ) is involved in regulating tumor growth and metastasis. However, the molecular mechanism underlying PLCγ-dependent tumor growth and metastasis of gastric adenocarcinoma and whether PLCγ may be a potential target for tumor therapy in human gastric adenocarcinoma are not yet well determined. Here, we investigated the role of PLCγ inhibition in tumor growth and metastasis of human gastric adenocarcinoma using BGC-823 cell line and a nude mouse tumor xenograft model. The results manifested that the depletion of PLCγ1 by the transduction with lentivirus-mediated PLCγ1 gene short-hairpin RNA (shRNA) vector led to the decrease of tumor growth and metastasis of human gastric adenocarcinoma *in vitro* and *in vivo*. Furthermore, the Akt/Bad, Akt/S6, and ERK/Bad signal axes were involved in PLCγ1-mediated tumor growth and metastasis of human gastric adenocarcinoma. Therefore, the abrogation of PLCγ1 signaling by shRNA could efficaciously suppress human gastric adenocarcinoma tumor growth and metastasis, with important implication for validating PLCγ1 as a potential target for human gastric adenocarcinoma.

## INTRODUCTION

Gastric cancer is a highly malignant tumor with a complex etiology that results in many patients being diagnosed after the disease has reaches an advanced stage or in the occurrence of relapse after curative surgery. In the effort to improve treatment, targeting specific molecules has emerged as a potentially effective approach that is less harmful to normal cells than currently available treatments [1]. The targeted molecules include signaling molecules, growth factor receptors and microRNAs [2, 3].

Phosphoinositide-specific phospholipase γ (PLCγ) is activated downstream of many receptor tyrosine kinases and growth factors [4]. PLCγ induces hydrolysis of phosphatidylinositol 4,5-bisphosphate (PtdIns(4,5)P2) to form the second messengers diacylglycerol (DAG) and inositol 1,4,5-trisphosphate (IP3), which in turn activate a number of signaling pathways to regulate the metabolism in many cell types, including cancer cells [4–7]. As an example, PLCγ contributes to metastasis of *in situ*-occurring mammary and prostate tumors [7]. PLCγ mediates high levels of glucose and insulin-induced cell proliferation and migration in MDA-MB-468 breast cancer and SW480 colon cancer cells *in vitro* [8]. Depletion of PLCγ expression or inhibition of its activity not only increases cisplatin-induced apoptosis but also suppresses the invasive ability of RhoGDI2-overexpressing SNU- 484 gastric cancer cells [9]. Thus PLCγ activity appears to support both tumor growth and metastasis.

Multiple signaling molecules mediate the effects of PLCγ. For example, STAT3 contributes to colorectal tumorigenesis through interaction with PLCγ1 [10]; the combined activation of PLCγ and MAPK is required for FGFR3-induced epithelial to mesenchymal transition (EMT) [11]; and FGF induces G2/M transition via the Akt/PLCγ1 axis in MDA-MB-231 breast cancer cells [12]. In this way, PLCγ1 plays a crucial role in fostering the growth and metastasis of some tumor types through interaction with other signal molecules [13], and may be a useful target for anti-tumor therapy.

Our previous study showed that PLCγ1 is strongly expressed in human gastric adenocarcinoma tissue, and that metastasis of human gastric adenocarcinoma depends in part on PLCγ1 expression [14]. Akt and PKCα are involved in mediating PLCγ signaling in gastric cancer cells [14, 15], but the molecular mechanism underlying PLCγ-dependent growth and metastasis of human gastric adenocarcinoma is not yet well determined.

BGC-823 cell line transduced with a lentivirus-mediated PLCγ1 gene short-hairpin RNA (shRNA) vector and a nude mouse xenograft model were used to investigate the mechanism by which PLCγ stimulates growth and metastasis of gastric adenocarcinoma. Our findings indicate that inhibiting PLCγ1 suppresses human gastric adenocarcinoma growth and metastasis and that the signaling molecules Akt, ERK, Bad and S6 are all involved. These findings suggest PLCγ1 may be a useful therapeutic target for the treatment of human gastric adenocarcinoma.

## RESULTS

### The effect of PLCγ1 shRNA expression on proliferation of human gastric adenocarcinoma cells

**Figure 1:**
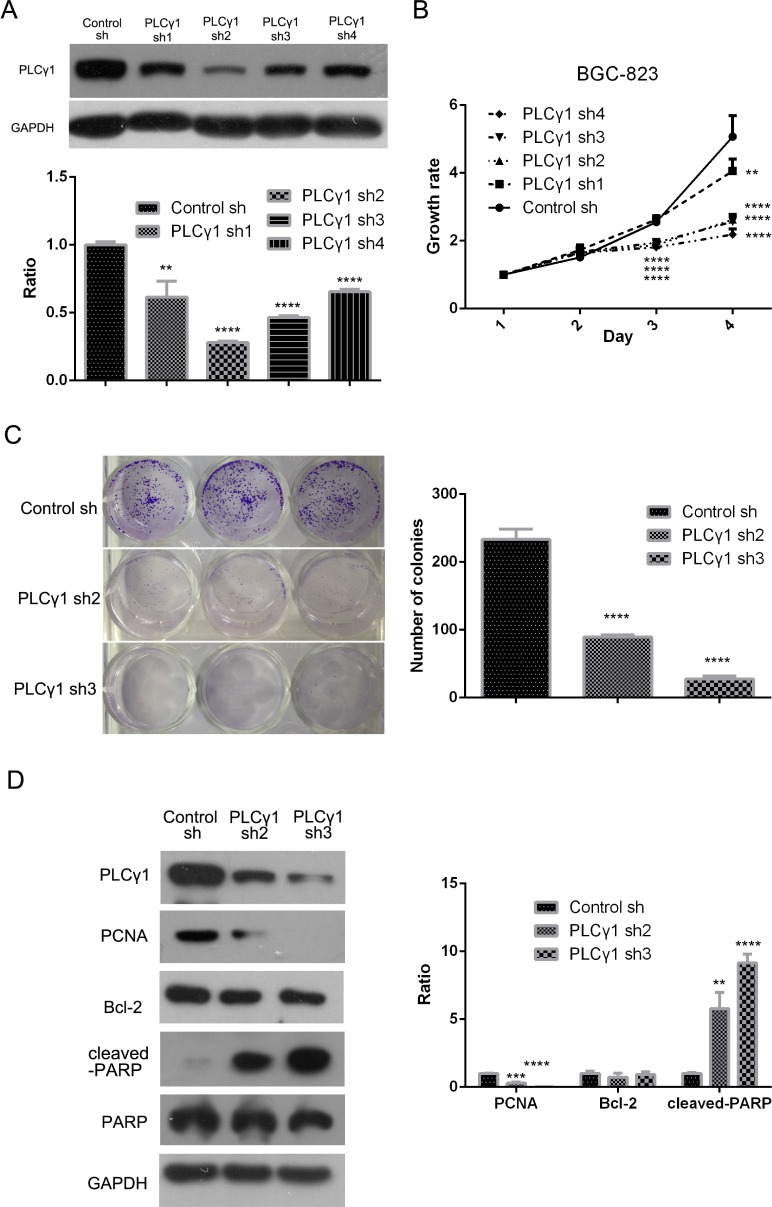
Lentivirus-mediated PLCγ1 shRNA could block proliferation in human gastric adenocarcinoma BGC-823 cells BGC-823 cell line of stable expressing PLCγ1shRNA was established with the transduction of four types PLCγ1shRNAs using a lentiviral transduction strategy. (**A**) The effect of PLCγ1shRNAs on the level of PLCγ1 protein was detected with Western blotting analysis as described in Materials and Methods. (**B**) The effect of PLCγ1shRNAs on cell growth rate was measured with MTT assay as described in Materials and Methods. (**C**) The effect of PLCγ1 shRNA2/3 on cloning formation was detected with Colony formation assay as described in Materials and Methods. (**D**) The levels of PCNA, cleaved-PARP, PARP, Bcl-2, PLCγ1, and GAPDH protein were detected with Western blotting analysis as described in Materials and Methods. Data are reported as means ± S.D. of three independent experiments (**P < 0.01, ***P < 0.01, ****P < 0.0001, *vs* respective control).

BGC-823 cells were transduced with four types of lentivirus-mediated PLCγ1 shRNA vector to establish stable cell lines expressing PLCγ1 shRNA. Figure 1A showed that all four PLCγ1 shRNA vectors effectively inhibited expression of PLCγ1 protein, but the efficacy of the PLCγ1 shRNA2/3 vectors was most prominent (**P < 0.01, ****P < 0.0001 *vs* control). Subsequent MTT and colony formation assays showed that depletion of PLCγ1 using shRNAs led to a decrease of growth rate (Figure 1B, **P < 0.01, ****P < 0.0001 *vs* control). The cloning efficiency was dramatically decreased in cells expressing PLCγ1 shRNA2/3 (Figure 1C, ****P < 0.0001 *vs* control). Furthermore, Western blot analysis indicated that the depletion of PLCγ1 led to a decrease in the level PCNA and an increase in the level of cleaved-PARP (Figure 1D, **P < 0.01, ***P < 0.001, ****P < 0.0001 *vs* control). On the other hand, Bcl-2 levels were unchanged. These results indicate that lentivirus-mediated PLCγ1 shRNAs suppress cell proliferation of human gastric adenocarcinoma cells.

### The effect of PLCγ1 on migration of human gastric adenocarcinoma cells

**Figure 2:**
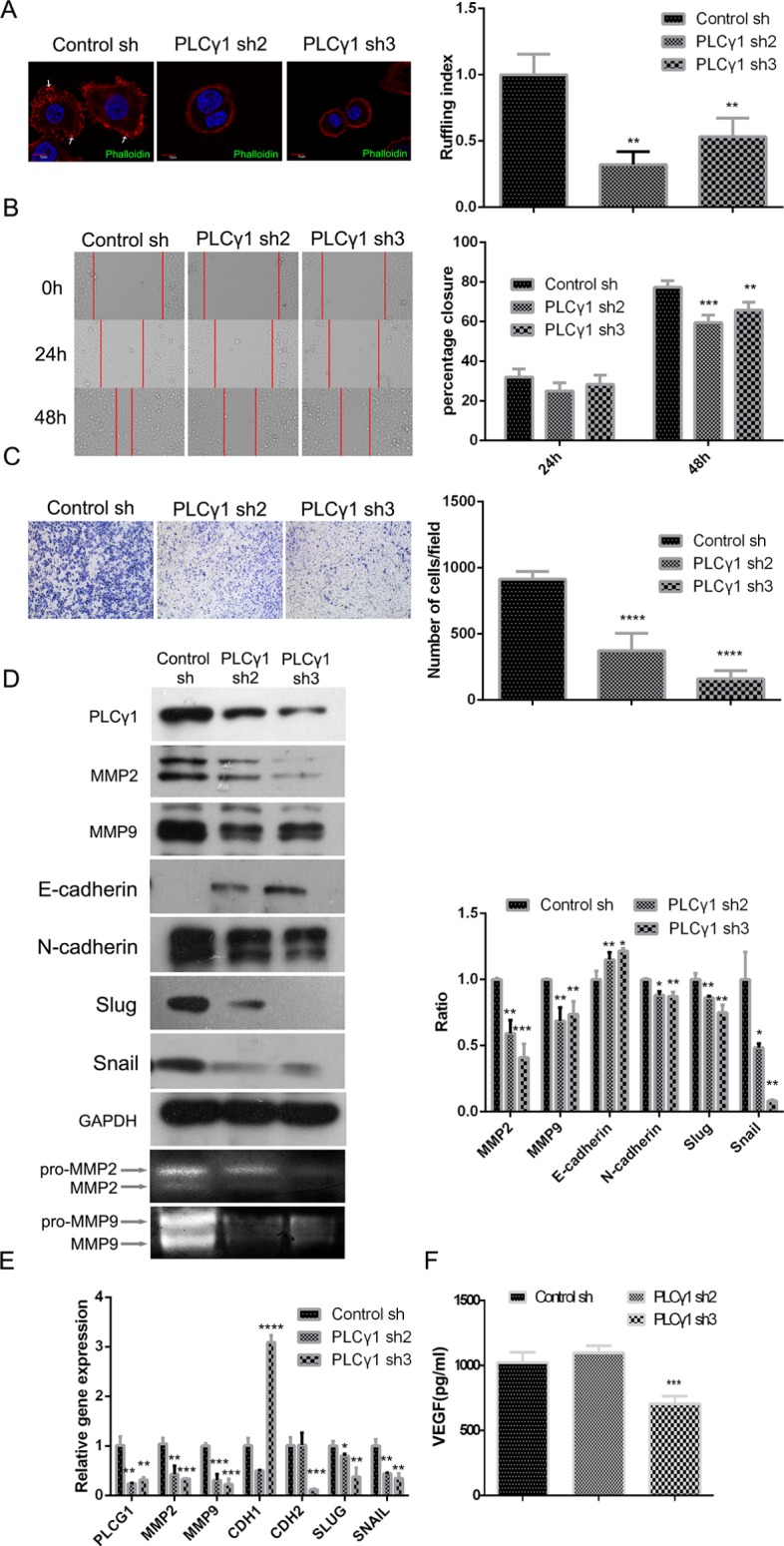
Lentivirus-mediated PLCγ1 shRNA could suppress migration in human gastric adenocarcinoma BGC-823 cells Cells were transduced with lentivirus-mediated PLCγ1 shRNA2/3 vectors. (**A**) The formation of membrane ruffles was detected using Ruffling assay as described in Materials and Methods. The cell nuclei were stained DAPI (blue) and the membrane ruffles were stained rhodamine-conjugated phalloidin (red). Scale bar = 10 μm. (**B** and **C**) The migration ability was measured using Transwell assay (B, magnification × 100) and Scratch assay (C, magnification × 400) as described in Materials and Methods. (**D**) The protein levels of MMP2, MMP9, E-cadherin, N-cadherin, snail, slug, and GAPDH were detected with Western blotting analysis, and the pro and active forms of MMP2/9 were observed using gelatin zymography assay as described in Materials and Methods. (**E**) The mRNA levels of PLCG1, MMP2, MMP9, CDHI, CDH2, SNAIL, SLUG, and GAPDH were detected using Real-time PCR analysis as described in Materials and Methods. (**F**) The level of VEGF in extracellular matrix was detected using ELISA as described in Materials and Methods. Data are reported as means ± S.D. of three independent experiments (*P < 0.05, **P < 0.01, ***P < 0.001, ****P < 0.0001, *vs* respective control).

To determine whether PLCγ1 is involved in cancer cell migration, we assessed the effects of PLCγ1 shRNA2/3 in ruffling, transwell, and scratch assays. As shown in Figure 2A, cells expressing PLCγ1 shRNA2/3 exhibited fewer membrane ruffles than control cells (**P < 0.01). The results of both scratch and transwell assays indicated that PLCγ1 depletion attenuated cell motility (Figure 2B and 2C, **P < 0.01, ***P < 0.001, ****P < 0.0001 *vs* control). Furthermore, the expression of main signal molecules involving in cell migration such as MMPs and EMT-related signal molecules was detected using Western blotting analysis, Gelatine zymography assay, and Real-time PCR analysis, respectively. The levels of MMP2/9, N-cadherin, snail, and slug protein were reduced by the depletion of PLCγ1, with the increase of E-cadherin protein (Figure 2D, *P < 0.05, **P < 0.01, ***P < 0.001, *vs* control). The secreted levels of MMP2/9 in extracellular matrix were also reduced in PLCγ1-transformed cells with PLCγ1 shRNA2/3 vectors (Figure 2D, lower panel). The depletion of PLCγ1 by shRNA2/3 led to the decrease in MMP2/9, SNAIL, SLUG, and CDH2 mRNA levels, with the increase in CDH1 mRNA level (Figure 2E, *P < 0.05, **P < 0.01, ***P < 0.001, *vs* control). Additionally, the level of vascular endothelial growth factor (VEGF) associated with tumor angiogenesis was then detected with ELISA. The results displayed that the depletion of PLCγ1 by shRNA3 suppressed VEGF expression in extracellular matrix (Figure 2F, ***P < 0.001, *vs* control). Therefore, lentivirus-mediated PLCγ1 shRNAs could suppress migration of human gastric adenocarcinoma cells.

### The depletion of PLCγ1 by shRNA suppresses tumor growth and metastasis in a nude mouse xenograft model of human gastric adenocarcinoma

**Figure 3:**
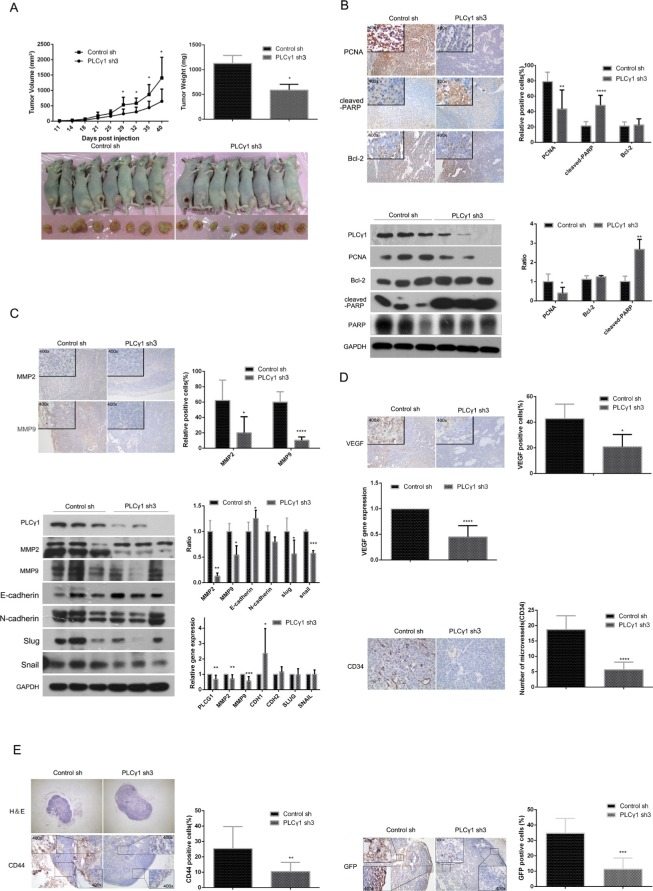
Depletion of PLCγ1 suppresses growth and metastasis of gastric adenocarcinoma in a nude mouse tumor xenograft model (**A**) Volume and weight of tumor samples from nude mice. (**B**) The protein levels of PCNA, cleaved-PARP, PARP, and Bcl-2 in the tumor samples were detected by Immunohistochemistry (Magnificationx100, x400) and Western blotting analyses as described in Materials and Methods. (**C**) The levels of MMP2 and MMP9 in the tumor samples were detected by Immunohistochemistry analysis as described in Materials and Methods (Magnificationx100, x400). The protein and mRNA levels of MMP2, MMP9, E-cadherin(CDH1), N-cadherin(CDH2), snail(SNAIL), and slug(SLUG) in the tumor samples were detected by Western Blotting and Real-time PCR analyses as described in Materials and Methods. (**D**) The protein levels of VEGF and CD34 and the mRNA level of VEGF in tumor samples were detected by Immunohistochemistry and Real-time PCR analysis as described in Materials and Methods. The number of microvessels was accounted under OLYPUS x41 microscope (Magnification x100, x400). (**E**) The lymphoid follicles in inguinal lymph nodes of nude mice were observed under OLYPUS x41microscope, and the protein levels of CD44 and GFP in inguinal lymph nodes of nude mice was detected by Immunohistochemistry analysis as described in Materials and Methods (Magnificationx40, x400). Data are reported as means ± S.D. of three independent experiments (*P < 0.05, **P < 0.01, ***P < 0.001, ****P < 0.0001, *vs* respective control).

To determine the potential role of PLCγ1 inhibition for the therapy of gastric adenocarcinoma, the status of tumor in a nude mouse model harboring tumor xenografts derived from BGC-823 cells transduced with PLCγ1 shRNA3 vector were investigated. The tumor volume after subcutaneous injection of PLCγ1-transformed BGC-823 cells for 29 days was suppressed (Figure 3A, left panel, *P < 0.05, *vs* control). The tumor weight was reduced in the group of PLCγ1-transformed BGC-823 cells (Figure 3A, right panel, *P < 0.05, *vs* control). Furthermore, the decrease in PCNA protein level and the increase in cleaved-PARP protein level were observed in tumor tissue using Immunohistochemistry and Western blotting analyses, while the level of Bcl-2 protein did not change (Figure 3B, **P < 0.01, ****P < 0.0001, *vs* control). Hence, the depletion of PLCγ1 by shRNA3 could suppress human gastric adenocarcinoma growth in a nude mouse tumor xenograft model.

In addition, MMP2/9 protein levels were reduced in tumor tissue using Immunohistochemistry analysis (Figure 3C, upper panel, *P < 0.05, ****P < 0.0001, *vs* control). Western blotting analysis showed that the depletion of PLCγ1 led to the decrease in MMP2/9, slug and snail protein levels, and the increase in E-cadherin protein level, while the level of N-cadherin protein did not change (Figure 3C, lower panel, *P < 0.05, **P < 0.01, ***P < 0.001, *vs* control). Similarly, the decrease in MMP2/9 mRNA levels and the increase in CDH1 mRNA level were observed in tumor tissue using Real-time PCR analysis (Figure 3C, the lowest panel, *P < 0.05, **P < 0.01, ***P < 0.001, *vs* control). On the other hand, the depletion of PLCγ1 by shRNA3 led to the decrease in CD34 (one of biomarkers of vascular density) and VEGF protein levels, and VEGF mRNA level in tumor tissue, indicating the involvement of PLCγ1 in the angiogenesis of tumor (Figure 3D, *P < 0.05, ****P < 0.0001, *vs* control). Figure 3E showed that the number of lymphoid follicles in inguinal lymph node, the level of CD44 that is one of import biomarkers in lymphatic node metastasis, and the level of GFP that is one of the tags of PLCγ1-transformed cells, were all reduced in tumor tissue, exhibiting the lymphatic node metastasis of gastric adenocarcinoma cells (**P < 0.01, ***P < 0.001, *vs* control). Overall, the depletion of PLCγ1 by shRNA could suppress tumor metastasis in a nude mouse xenograft model of human gastric adenocarcinoma.

### The involvement of Akt, ERK, mTOR, Bad, and S6 in PLCγ1-mediated proliferation and migration of human gastric adenocarcinoma cells

**Figure 4:**
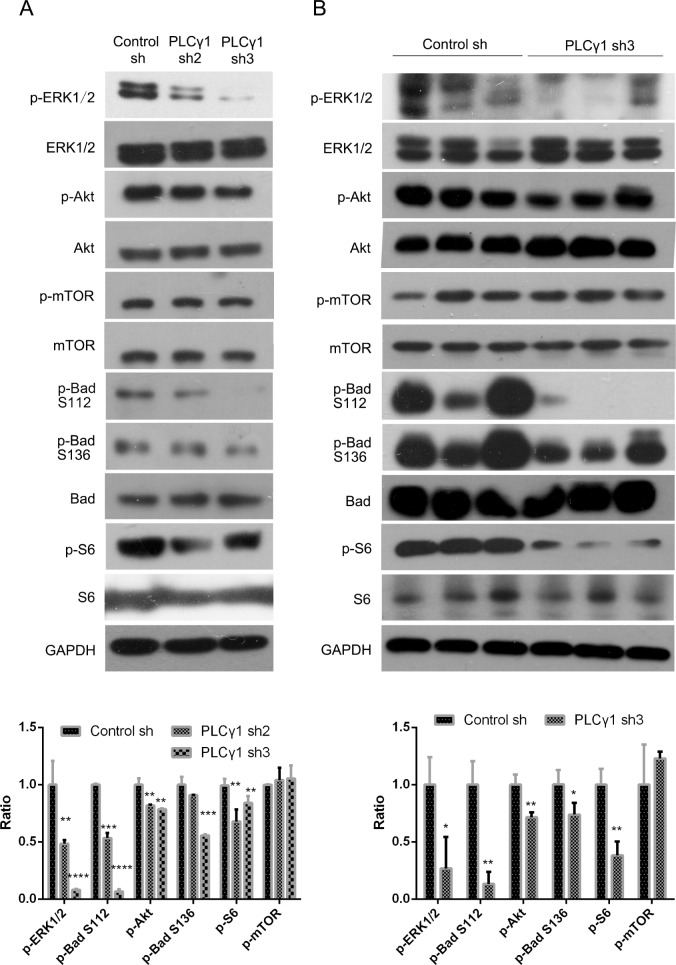
Effect of PLCγ1 inhibition on signal molecules associated with tumor growth and metastasis of human gastric adenocarcinoma (**A**) Cells were transduced with lentivirus-mediated PLCγ1shRNA2/3 vectors. The protein levels of Akt, p-Akt, ERK1/2, p-ERK1/2, mTOR, p-mTOR, S6, p-S6(Ser235/236), Bad, p-Bad (Ser112), p-Bad (Ser136), PLCγ1, and GAPDH were detected with Western blotting analysis as described in Materials and Methods. (**B**) A nude mouse model harboring tumor xenografts derived from BGC-823 cells transduced with PLCγ1-shRNA3 vector was constructed. The protein levels of Akt, p-Akt, ERK1/2, p-ERK1/2, mTOR, p-mTOR, S6, p-S6(Ser235/236), Bad, p-Bad (Ser112), p-Bad (Ser136), PLCγ1, and GAPDH in the tumor samples were detected by Western blotting analysis as described in Materials and Methods. Data are reported as means ± S.D. of three independent experiments (*P < 0.05, **P < 0.01, ***P < 0.001, ****P < 0.0001, *vs* respective control).

To investigate the regulatory mechanism of PLCγ1 in cell proliferation and migration, the effect of PLCγ1 shRNA on the expression of some important signaling molecules, such as Akt, ERK, mTOR, Bad, S6, MMP, and EMT-related molecules, was detected using Western blotting analysis. Figure 4A showed that the depletion of PLCγ1-shRNA2/3 down-regulated the levels of p-Akt, p-ERK1/2, p-Bad (Ser112), p-Bad (Ser136), and p-S6 (Ser235/236), without the alteration of Akt, ERK1/2, S6, and Bad levels in *in vitro* PLCγ1-transformed BGC-823 cells (*P < 0.05, **P < 0.01, ***P < 0.001, ****P < 0.0001, *vs* control). The depletion of PLCγ1 by shRNA3 led to the decrease in p-Akt, p-ERK1/2, p-Bad (Ser112), p-Bad (Ser136), and p-S6 (Ser235/236) levels in tumor tissue of human gastric adenocarcinoma xenografts *in vivo* (Figure 4B, *P < 0.05, **P < 0.01, ***P < 0.001, *vs* control). The levels of mTOR and p-mTOR did change neither *in vitro* nor *in vivo*. These results indicated that the regulatory mechanism of PLCγ1 on tumor growth and metastasis of human gastric adenocarcinoma was associated with the phosphorylation of Akt, ERK1/2, Bad, and S6 signaling molecules *in vitro* and *in vivo*.

## DISCUSSION

Our findings indicate that the depletion of PLCγ1 by the transduction with lentivirus-mediated PLCγ1 shRNA vector led to the suppression of tumor growth and metastasis in human gastric adenocarcinoma *in vitro* and *in vivo*. Furthermore, the phosphorylation of Akt, ERK, Bad, and S6 signaling molecules was involved in PLCγ1-mediated tumor growth and metastasis of human gastric adenocarcinoma cells. The data suggest that the abrogation of PLCγ1 signaling by shRNA could efficaciously suppress tumor growth and metastasis of human gastric adenocarcinoma through Akt/Bad, ERK/Bad, and Akt/S6 signal axes, implying that PLCγ1 is a potential target for human gastric adenocarcinoma.

Inhibiting PLCγ1 has been known to suppress cell proliferation in several types of tumor, including digestive system tumors [9, 10, 16]. Furthermore, combined inhibition of PLCγ1 and c-Src abrogates EGFR-mediated head and neck squamous cell carcinoma invasion [17]. Targeting PLCγ1 by way of a hammerhead ribozyme to PLCγ1 reduces the invasive phenotype of prostate cancer [18]. The above-mentioned studies exhibit that the abrogation of PLCγ1 could efficaciously block both cell proliferation and migration. Our data also displayed that the depletion of PLCγ1 by shRNA suppressed cell proliferation and migration of human gastric adenocarcinoma cells *in vitro* and *in vivo*. Therefore, it is suggested that the blockade of PLCγ1 could be a therapeutic approach for preventing tumor growth and metastasis in human gastric adenocarcinoma. Moreover, in previous studies, the deficiency of the selectivity of the pharmacological inhibitor of PLCγ1 (U73122) was always to be neglected [19]. Here, the depletion of PLCγ1 by shRNA exhibited stronger selectivity than that of pharmacological mean. Combined with other studies [20, 21], the shRNA technique indeed maybe an efficacious approach for tumor therapy.

Several lines of evidence have demonstrated the involvement of PLCγ1 in angiogenesis. As an example, PLCγ1 is required downstream of VEGF during arterial development [22]. Furthermore, the absence of erythrogenesis and vasculogenesis in PLCγ1-deficient mice is detected [23]. As angiogenesis is the requirement for tumor metastasis, we detected the effect of PLCγ1 shRNA on the two biomarkers of angiogenesis, VEGF and CD34. Our data indicated that the levels of VEGF and CD34 were reduced by PLCγ1 shRNA, consistent with recent studies. Brain-derived neurotrophic factor increases VEGF expression and enhances angiogenesis in human chondrosarcoma cells through a signal transduction including TrkB receptor, PLCγ, and PKCα [24]. Trans presentation of VEGF and sustained PLCγ activation might have contributed to the reduced vascular density in matrigel tumors [25]. Therefore, we suggested that the inhibition of PLCγ1 could suppress tumor angiogenesis in human gastric adenocarcinoma. In addition, EMT is one of pivotal elements in tumor metastasis. It has been well known that a variety of growth factors, such as FGF and EGF, could cooperate with multiple pathways in the induction of EMT [11, 12, 16, 24]. As an example, FGFR1 activation promotes EMT in rodent models of breast and prostate cancer [26, 27]. In view of the relationship between growth factors and PLCγ activation [4–7], and our data of the alternation of EMT associated signal molecules at protein and mRNA levels in BGC-823 cells with the transduction by PLCγ1 shRNA vector, we proposed that PLCγ has a promoting effect on the EMT progression in human gastric adenocarcinoma. Moreover, Tomlinson DC et al. also demonstrated the involvement of PLCγ in the FGFR1-induced EMT progression in UC cells lines [11]. Overall, it is confirmed that the inhibition of PLCγ1 could suppress tumor metastasis through blocking angiogenesis and EMT progression in human gastric adenocarcinoma. The molecular mechanism of PLCγ1 regulating angiogenesis and EMT in human gastric adenocarcinoma needs further to be studied.

Akt and ERK signal molecules have been well known to regulate cell proliferation and migration of human gastric cancer cells [28–30]. Especially, it has been reported that Akt and ERK could regulate the identical cell events in some cancer cells. For example, regenerating gene Iα protein that is overexpressed in a subset of gastric cancers promotes growth and angiogenesis through activation of the ERK and Akt signaling pathways in HUVEC cells [31]. Plexin-B1 silencing inhibits the phosphorylation of Akt and ERK to reduce the growth, proliferation, migration, and invasion of A431 cells [32]. Afatinib resistance in non-small cell lung cancer involves the PI3K/AKT and MAPK/ERK signalling pathways [33]. The de-ubiquitinase Ubiquitin C-terminal hydrolase-L1(UCHL1) promotes gastric cancer metastasis via the Akt and ERK1/2 pathways [34]. Licochalcone A induces human gastric cancer BGC-823 cell apoptosis by regulating ROS-mediated MAPKs and PI3K/Akt signaling pathways [35]. In accordance with the above-mentioned studies, the inhibition of PLCγ1 suppressed human gastric adenocaecinoma growth and metastasis via Akt and ERK signaling pathways. On the other hand, the involvement of Akt and ERK signaling pathways in PLCγ1-mediated tumor growth and metastasis implied the existence of the crosstalk of PLCγ1 with Akt or ERK in human gastric adenocarcinoma, which has been also demonstrated in some cancer cells. As an example, Akt interaction with PLCγ regulates the G(2)/M transition triggered by FGF receptors from MDA-MB-231 breast cancer cells [12]. PLCγ1 could activate ERK1/2 through the PLCγ1-PKCα-B-Raf pathway in VEGF-treated endothelial cells [36]. Therefore, PLCγ regulates human gastric adenocarcinoma growth and metastasis through interacting with Akt or ERK. In addition, we demonstrated that Akt could phosphorylate its substrates, including BAD at Ser-136 and S6 at Ser235/236, to promote cell proliferation and protein synthesis of human gastric adenocarcinoma cells, as described in the conventional regulatory mechanism of Akt [37, 38]. ERK could phosphorylate BAD at Ser-112 via p90RSK and promotes cell proliferation of human gastric adenocarcinoma cells, similar to its classical regulatory mechanism [39]. The data indicated the existence of Akt/Bad, ERK/Bad, and Akt/S6 axes in PLCγ1-mediated tumor growth, metastasis and angiogenesis in human gastric adenocarcinoma.

In conclusion, the inhibition of PLCγ1 by the transduction with Lentivirus-mediated PLCγ1 gene short-hairpin RNA vector led to the decrease of tumor growth and metastasis of human gastric adenocarcinoma *in vivo* and *in vitro*. Furthermore, Akt/Bad, ERK/Bad, and Akt/S6 axes were involved in PLCγ1-mediated cell proliferation and migration of human gastric adenocarcinoma cells. The data suggest that inhibiting PLCγ1 could suppress human gastric adenocarcinoma growth and metastasis, indicating that PLCγ1 is a potential target for human gastric adenocarcinoma.

## MATERIALS AND METHODS

### Reagents and antibodies

Antibodies against PLCγ1, Akt, p-Akt (Ser473), ERK1/2, p-ERK1/2 (Thr202/Tyr204), mTOR, p-mTOR (Ser2481), S6, p-S6 (Ser235/236), Bad, p-Bad (Ser112or 136), Bcl-2, GFP, slug, and snail were purchased from Cell Signaling Technology Inc. (Beverly, MA, USA). PCNA, PARP, cleaved-PARP, MMP9, and GAPDH were purchased from Abcam (Cambridge, MA, USA). MMP2, E-cadherin, and N-cadherin were purchased from Santa Cruz Biotechnology (Santa Cruz, CA, USA). VEGF, CD34, and CD44 were purchased from BOSTER (Wuhan, China). Other reagents were of the highest grade commercially available.

### Cell culture

The human gastric cancer cell line, BGC-823, was obtained from the Shanghai Institute of Cell Biology, Chinese Academy of Sciences, Shanghai, China, and was maintained in DMEM medium supplemented with 10% fetal bovine serum (FBS), 100 U/mL penicillin, and 100 μg/mL streptomycin, at 37°C in a water-saturated atmosphere of 5% CO^2^.

### Plasmid construction and transduction

Lentiviral-mediated shorthairpin RNA (shRNA) targeting PLCγ1

(Sh1: 5′ CcgggcCATTGACATTCGTGAAATTctc gagAATTTCACGAATGTCAATGgcTTTTTg3′. Sh2: 5′ CcggccAGATCAGTAACCCTGAATTctcgagAATTCAG GGTTACTGATCTggTTTTTg3′. Sh3: 5′ CcggccTGTGAA CCACGAATGGTATctcgagATACCATTCGTGGTTCAC AggTTTTTg3′. Sh4: 5′ CcggccAGGGAAACAAAGTTTA CATctcgagATGTAAACTTTGTTTCCCTggTTTTTg3′.) was purchased from Gene Chem (Shanghai, China). The different shPLCγ1vectors were transduced into BGC-823 cells using a lentiviral transduction strategy, respectively. ShPLCγ1 stable cell lines were obtained under the pressure of puromycin (2 μg/ml, BioVision, Inc., CA, USA). The level of PLCγ1 protein was detected with Western blotting analysis prior to the other experiments.

### Real-time PCR analysis

Total RNA in cells and tissues was extracted using Trizol (Invitrogen, CA, USA). The amount and quality of the extracted RNA was assessed by spectrophotometry using NanoDrop 2000 (NanoDrop Technologies Inc., DE, USA). cDNA synthesis was performed with 1 μg of total RNA at 37°C for 15min using the Primescript RT Master Mix Kit (Takara, Dalian, China), and subsequently diluted 10-fold. Real-time PCR analysis was performed using the ABI StepOnePlus Sequence Detection System v2.1 (Applied Biosystems, Singapore) with SYBR Premix Ex Taq II Kit (Takala, Dalian, China). Results were normalized to GAPDH and analyzed using SDS software v2.1 according to previous study [40]. The following primer was used in quantitative PCR for measuring gene expression relative to GAPDH (Table 1).

**Table 1:** Primers in quantitative PCR

Gene name	Primer sequence (5′→3′)
GAPDHOMIM *138400	Forward 5′-GGAAGGTGAAGGTCGGAGTCA-3′ Reverse 5′-GTCATTGATGGCAACAATATCCACT-3′
hCDH1 OMIM *192090	Forward 5′-TTGCACCGGTCGACAAAGGAC-3′ Reverse 5′-TGGATTCCAGAAACGGAGGCC-3′
hCDH2 OMIM *114020	Forward 5′-TGTCGGTGACAAAGCCCCTG-3′ Reverse 5′-AGGGCATTGGGATCGTCAGC-3′
hSNAILOMIM *612741	Forward 5′-CTGGGTGCCCTCAAGATGCA-3′ Reverse 5′-CCGGACATGGCCTTGTAGCA-3′
hSLUG OMIM *602150	Forward 5′-TACCGCTGCTCCATTCCACG-3′ Reverse 5′-CATGGGGGTCTGAAAGCTTGG-3′
hPLCG1 OMIM*172420	Forward 5′-TGTCCCACAGACCAACGC-3′ Reverse 5′-ATTCCGCTTCCGCACCAG-3′
hMMP2OMIM *120360	Forward 5′-AGTAAACAGCAAGAGAACCT Reverse 5′-AACAGATGCCACAATAAAGC
hMMP9OMIM *120361	Forward 5′-ACTACTGTGCCTTTGAGTC Reverse 5′-TACTTCCCATCCTTGAACAA
hVEGF OMIM +192240	Forward 5′-CTTGCCTTGCTGCTCTACCT Reverse 5′-ACGCGAGTCTGTGTTTTTGC

### MTT assay

Cells infected by lentivirus were seeded in 96-well plates (1 × 10^4^ cells/well) and cultured for the indicated time. The number of viable cells was detected using 3– (4,5–Dimethylthiazol-2–y)–2,5-diphenyl-tetrazolium bromide (MTT) assay as described previously [14, 41].

### Determination of human VEGF by ELISA

The cell culture supernatant was collected and added to each ELISA plate well pre-coated with anti-human VEGF polyclonal antibody. The level of VEGF in the culture supernatant was then measured by the human VEGF ELISA kit (Neobioscience, Shenzhen, China) according to the manufacturer's instruction and previous study [42].

### Colony formation assay

Cells transduced with different shRNA vectors were plated in 6-well plate for the indicated time. The colonies formed from each cell were fixed with 100% methanol, stained with crystal violet, and counted according to previous study [43].

### Scratch assay

As described in previous studies [14, 44], cells were seeded onto 6-well plates and two centerlines were marked on the upside of each well along its horizontal axis, to designate the loci at which images would be acquired at each time point. Vertical linear scratches were introduced into the cell monolayers using a 10 μL sterile pipette tip. Each well received 2 scratches. Images at 40x magnification were acquired at 0, 24, and 48 h after scratching at each intersection of the scratch wound (vertical defect). For each time point, 4 measurements were taken per well in each of 3 wells, and the average of the horizontal width of the linear defect in pixels was calculated using the Image-Pro Plus 6.0 system. The mean percentage closure was calculated by compared with time 0.

### Transwell assay

Cell migration was performed in Transwell chambers (Corning Inc., Corning, USA) as described in previous studies [14, 45]. Briefly, cells in serum-free DMEM were placed into the upper chambers of Transwell inserts set within wells with 8 μm pore filters, and incubated at 37°C for 12 h. The migrated cells on the lower membrane surface were fixed in methanol and stained with 0.1% Giemsa stain. Eight microscope fields from each Transwell chamber were randomly selected, and cells adhering to the undersurface of the filter were imaged and counted using an Olympus BX41 microscope equipped with a digital camera (Olympus, Tokyo, Japan).

### Gelatine zymography assay

The assay was conducted according to published protocol [14, 46]. Briefly, protein extracts in the conditioned media were electrophoresed on 6% SDS polyacrylamide gels containing 1 mg/ml of gelatine (Bio Basic Inc., Markham, Ontario, Canada). The gels were then washed twice for 30 min in 2.5% Triton X-100 at room temperature, and incubated for 48 h at 37°C in incubation buffer (50 mM Tris-HCl (pH 7.5), 5 mM CaCl2, 150 mM NaCl, 1 μM ZnCl2, and 0.2% Brij35), followed with the staining of 0.25% (w/v) Coomassie brilliant blue R-250 for 1 h and de-stained in de-staining buffer (10% acetic acid and 50% methanol).

### Ruffling assay

Cells were seeded on glass coverslips in 6-well plates for 24 h, rinsed with PBS once, fixed in 4% paraformaldehyde for 10 min, and then washed with PBS three times. After 5 min permeation with 0.5% Triton X-100, cells were incubated with Rhodamine-conjugated Phalloidin (Cytoskeleton Inc., Denver, CO, USA) for 30 min at room temperature, before being stained with DAPI for 30 sec. Cells were observed and photographed using Confocal microscopy [13, 14].

### Western blotting analysis

Protein extracts were electrophoresed on 8–12% denaturing gel and electroblotted onto nitrocellulose membrane. The membrane was incubated with various antibodies as required at 4°C overnight, followed by the addition of the corresponding secondary antibody at room temperature for 1 h. An ECL kit (Pierce, Rockford, IL) was used to detect the antibody reactivity [41].

### Tumor xenograft model

Thirty two 6-week-old female BALB/Cnu/nu nude mice were purchased from Shanghai Slac Laboratory Animal Co. Ltd. (Shanghai, China). All animal studies were conducted according to the regulations of the Institutional Animal Care and Use Committee protocol. This study was approved by the Committee on the Ethics of Animal Experiments of the University of Xiamen (ID No.20110916). Animals bearing tumors were randomly assigned to 4 groups, 200 μl relevant stable cells (Control-sh, PLCγ1-sh3, 2 × 10^6^/mouse) in PBS were subcutaneously injected into the right hind leg of mouse, with 8 mice per group [28, 47]. Tumor volume and animal weight were measured every 3–4 days. All animals were killed since injected for 40 days. The mRNA and protein levels of relative signal molecules in these subcutaneous tumors and the inguinal lymph node were detected with RT-PCR, Western blotting analysis, and Immunohistochemistry. The morphology of inguinal lymph node was examined with H & E staining.

### Immunohistochemistry analysis

The fresh samples were fixed in 4% paraformaldehyde for 48 h, and then paraffin-embedded for further routine histological preparation. Four-micrometer-thick sections were deparaffinized in xylene and rehydrated in graded alcohols and distilled water, followed with immunohistochemistry staining according to the manufacturer's instructions (Maixin-Bio, Fuzhou, China) and previous study [14, 26]. The average optical density of positive cells was measured and analyzed by ImagePro Plus 6.0 system [14, 26].

### Statistical analysis

Experimental data were formulated as the means ± S.D. of triplicate independent samples. The differences between the groups were examined for statistical significance using *t*-test with GraphPad Prism, version 5(GraphPad Software, Inc., San Diego, CA. USA). The criterion for statistical significance was *P* < 0.05.
